# New York Mortuary Statistics

**Published:** 1857-09

**Authors:** 


### NEW YORK MORTUARY STATISTICS.

| Year. | Population. | Total Deaths. | Foreign Deaths. | Immigrants. |
|---|---|---|---|---|
| 1847 | 400,000 | 15,788 | 5,224 | |
| 1848 | | 15,919 | 4,490 | 189,176 |
| 1849 | Cholera. | 23,773 | 8,381 | 220,790 |
| 1850 | 515,394 | 16,978 | 5,060 | 212,796 |
| 1851 | | 22,024 | 6,351 | 289,601 |
| 1852 | | 21,601 | 6,492 | 300,992 |
| 1853 | | 22,702 | 7,104 | 284,945 |
| 1854 | Cholera. | 28,568 | 9,948 | 319,223 |
| 1855 | 629,810 | 23,042 | 6,103 | 136,233 |
| 1856 | 700,000 | 21,658 | | 141,672 |

During 1855, the fewest deaths occurred in November and December, being 2918; the highest mortality was in July and August, being 5121.

The fewest colored persons died in June, while three times as many died in February, indicating that a warm climate suits colored persons the best.

Most consumptive persons died in March—287; the fewest in May—141.

The most births occurred in February—1402; the fewest in November—944.

The most marriages were in April—459; the fewest in August—279.

Of the five chief destroyers, scarlet fever was 1035; cholera infantum, 1135; wasting, 1563; convulsions, 1895; consumption, 2635.

